# Improved tactile speech robustness to background noise with a dual-path recurrent neural network noise-reduction method

**DOI:** 10.1038/s41598-024-57312-7

**Published:** 2024-03-28

**Authors:** Mark D. Fletcher, Samuel W. Perry, Iordanis Thoidis, Carl A. Verschuur, Tobias Goehring

**Affiliations:** 1 University of Southampton Auditory Implant Service, University of Southampton, University Road, Southampton, SO17 1BJ UK; 2 Institute of Sound and Vibration Research, University of Southampton, University Road, Southampton, SO17 1BJ UK; 3 School of Electrical and Computer Engineering, Aristotle University of Thessaloniki, 54124 Thessaloniki, Greece; 4 MRC Cognition and Brain Sciences Unit, University of Cambridge, 15 Chaucer Road, Cambridge, CB2 7EF UK

**Keywords:** Translational research, Auditory system, Sensorimotor processing, Sensory processing

## Abstract

Many people with hearing loss struggle to understand speech in noisy environments, making noise robustness critical for hearing-assistive devices. Recently developed haptic hearing aids, which convert audio to vibration, can improve speech-in-noise performance for cochlear implant (CI) users and assist those unable to access hearing-assistive devices. They are typically body-worn rather than head-mounted, allowing additional space for batteries and microprocessors, and so can deploy more sophisticated noise-reduction techniques. The current study assessed whether a real-time-feasible dual-path recurrent neural network (DPRNN) can improve tactile speech-in-noise performance. Audio was converted to vibration on the wrist using a vocoder method, either with or without noise reduction. Performance was tested for speech in a multi-talker noise (recorded at a party) with a 2.5-dB signal-to-noise ratio. An objective assessment showed the DPRNN improved the scale-invariant signal-to-distortion ratio by 8.6 dB and substantially outperformed traditional noise-reduction (log-MMSE). A behavioural assessment in 16 participants showed the DPRNN improved tactile-only sentence identification in noise by 8.2%. This suggests that advanced techniques like the DPRNN could substantially improve outcomes with haptic hearing aids. Low-cost haptic devices could soon be an important supplement to hearing-assistive devices such as CIs or offer an alternative for people who cannot access CI technology.

## Introduction

Hearing-assistive devices often fail to effectively extract speech from background noise^1^. Users therefore often struggle to follow conversations in challenging listening environments, such as busy classrooms, cafes, and offices. Cochlear implant (CI) users tend to have particular difficulty in these scenarios^2^. Several recent studies have shown that supplementing the electrical CI signal by providing speech information through haptic stimulation (“electro-haptic stimulation”^3^) can improve speech-in-noise performance^3–9^. There is also evidence that these haptic hearing aids can be effective as standalone sensory substitution devices. These could be used to support the many millions of people who could benefit from a CI but cannot access the technology due to medical constraints or limitations in healthcare provision^5,10–14^. For haptic hearing aids to be effective, either for sensory substitution or augmentation, audio-to-haptic conversion strategies that are robust to background noise must be developed.

Some previous studies have avoided the issue of noise robustness by converting the clean speech signal to haptic stimulation, rather than the speech-in-noise signal that would be received by the microphone of a haptic hearing aid^6–8^. While these studies, which have shown substantial benefits to speech-in-noise performance, provide a proof-of-concept, they leave unaddressed a major obstacle in the translation of laboratory benefits to the real world. Other studies have converted the speech-in-noise signal to haptic stimulation and have also shown considerable benefits to speech recognition^3,4,9^. The method used to reduce the impact of noise in these studies was envelope expansion, which enhances high intensity parts of the haptic signal. While envelope expansion is effective when speech is considerably more intense than background noise^3,4,9^, it breaks down at more challenging (low or negative) signal-to-noise ratios (SNRs)^3,4^. If haptic signal extraction strategies that are robust in these more challenging listening scenarios can be developed, this would substantially widen the group of people who could benefit from haptic hearing aids.

One advantage of haptic hearing aids compared to other hearing-assistive devices, such as CIs or acoustic hearing aids, is that they are typically worn on the body (e.g., the wrist) rather than the head. They therefore have significantly more form-factor flexibility and design space available. This allows the use of larger, more powerful batteries and microprocessors that can deploy more sophisticated noise-reduction techniques^15^. The current study explored whether an advanced real-time-feasible noise-reduction method, using a recurrent neural network, can effectively extract the haptic speech signal from background noise at challenging SNRs and improve tactile speech perception.

Noise-reduction or “speech enhancement” techniques, aim to enhance the intelligibility and quality of speech in background noise. Traditional approaches using statistical models, such as log-spectra amplitude estimators, have failed to improve the intelligibility of speech in noisy environments commonly encountered in the real world, which contain competing speech and other fluctuating sounds^16^. Recently, data-driven machine-learning methods have revolutionised noise reduction, particularly for extracting speech in these more challenging scenarios. Such methods, which are usually based on deep learning (including variants of artificial neural networks), can substantially improve speech intelligibility for normal-hearing listeners^17^, listeners with hearing loss^18,19^, and CI users^20,21^. One of the most effective deep-learning noise-reduction methods developed in this rapidly advancing field is the dual-path recurrent neural network (DPRNN)^22^. The DPRNN operates on the long-term speech signal while retaining high temporal and spectral resolution. Critically, it also has excellent generalisation to unseen talkers and background noises.

Conventional recurrent neural networks are ineffective at modelling long sequences due to optimization issues, such as vanishing and exploding gradients^23^. The long short-term memory (LSTM) method was developed to overcome these deficiencies^24,25^. The DPRNN architecture utilizes residual LSTM blocks with layer-normalized alternating global and local processing paths to improve performance over a single LSTM layer or multiple LSTM layers stacked together. The DPRNN uses an encoder-masker-decoder architecture and recent work has shown that, across several metrics, it can outperform other state-of-the-art networks, such as ConvTasNet^22^. Furthermore, the DPRNN is more efficient than alternative cutting-edge architectures, such as the Dual-Path Transformer Neural Network^26^ and the Dual-Path Convolution Recurrent Network^27^, regarding the number of trainable parameters and the computational load.

The current study assessed whether a quasi-causal real-time feasible DPRNN approach could improve tactile speech-in-noise performance. This DPRNN was parameterised to fulfil processing latency requirements for real-time applications by using causal normalization methods and only 5.75-ms of future information. The DPRNN architecture integrates bi-directional LSTM units for local processing within each 5.75-ms window and uni-directional LSTM units for sequential processing across these windows to avoid further delay. The algorithmic delay is well below the processing latency of around 20–30 ms that would usually be considered tolerable for hearing-assistive devices, such as hearing aids and CIs^28^.

For audio-to-tactile conversion, the audio was processed with the quasi-causal DPRNN before being converted to vibro-tactile stimulation on the wrist using a previously developed tactile vocoder approach^3,4,9,10,14^. The tactile vocoder filters the audio into several frequency bands, and the amplitude envelope from each band is used to modulate the amplitude of one of several vibro-tactile tones. The tactile vocoder approach has been shown to be effective for transferring phoneme information^10,14^ and improving speech-in-noise performance for CI users^3,4,9^.

The quasi-causal DPRNN was assessed both objectively and behaviourally. The objective assessment aimed to establish whether the quasi-causal DRPNN could effectively extract speech from multi-talker noise and outperform traditional noise-reduction methods. The multi-talker noise used was representative of environments that listeners with hearing loss typically find most challenging, with competing speech and other fluctuating and transient sounds. The behavioural assessment tested whether objectively measured denoising of the tactile speech in multi-talker noise signal by the DPRNN translated into improvements in tactile speech identification. In objective testing, the quasi-causal DPRNN was compared to log MMSE^29^, which is a leading traditional noise-reduction method^30^. Note that the expander approach used in previous studies with tactile speech in noise is not suitable for an objective comparison such as this, because it intentionally alters the speech signal. It therefore cannot be directly compared to these other noise-reduction methods for which clean speech serves as a reference. Furthermore, as discussed, the expander approach has been shown to be ineffective at the challenging SNRs that this study focuses on. In addition to log MMSE, the quasi-causal DPRNN was compared to causal and non-causal implementations (all trained using the same data) to establish the effect of including future information.

In the objective assessment, the scale-invariant signal-to-distortion ratio (SI-SDR) of the tactile vocoder amplitude envelopes was compared for speech in either a multi-talker or a stationary (speech-shaped) noise. The speech and noise stimuli used in the objective assessment had not been used to train the DPRNNs. The assessment was done for a range of SNRs (− 2.5 dB, 2.5 dB, and 7.5 dB). Additional SI-SDR and eSTOI^31^ scores were also extracted for the audio (without tactile vocoding) to allow a comparison between the noise reduction methods used in the current study and those used in other hearing studies. These two measures are widely employed to assess speech processing algorithms, with eSTOI used to predict speech intelligibility for human listeners.

In the behavioural assessment, tactile sentence identification with and without the quasi-causal DPRNN was measured both in quiet and in multi-talker noise (the same that was used in objective testing). In noise, the SNR was set to 2.5 dB (though note that this is equivalent to around 0 dB SNR in many previous studies; see “Methods”). This represents one of the more challenging SNRs that listeners with hearing loss encounter in daily life^32,33^ and is an SNR at which most CI users have no appreciable speech intelligibility^3,9^.

## Results

### Objective assessment

**Figure 1:**
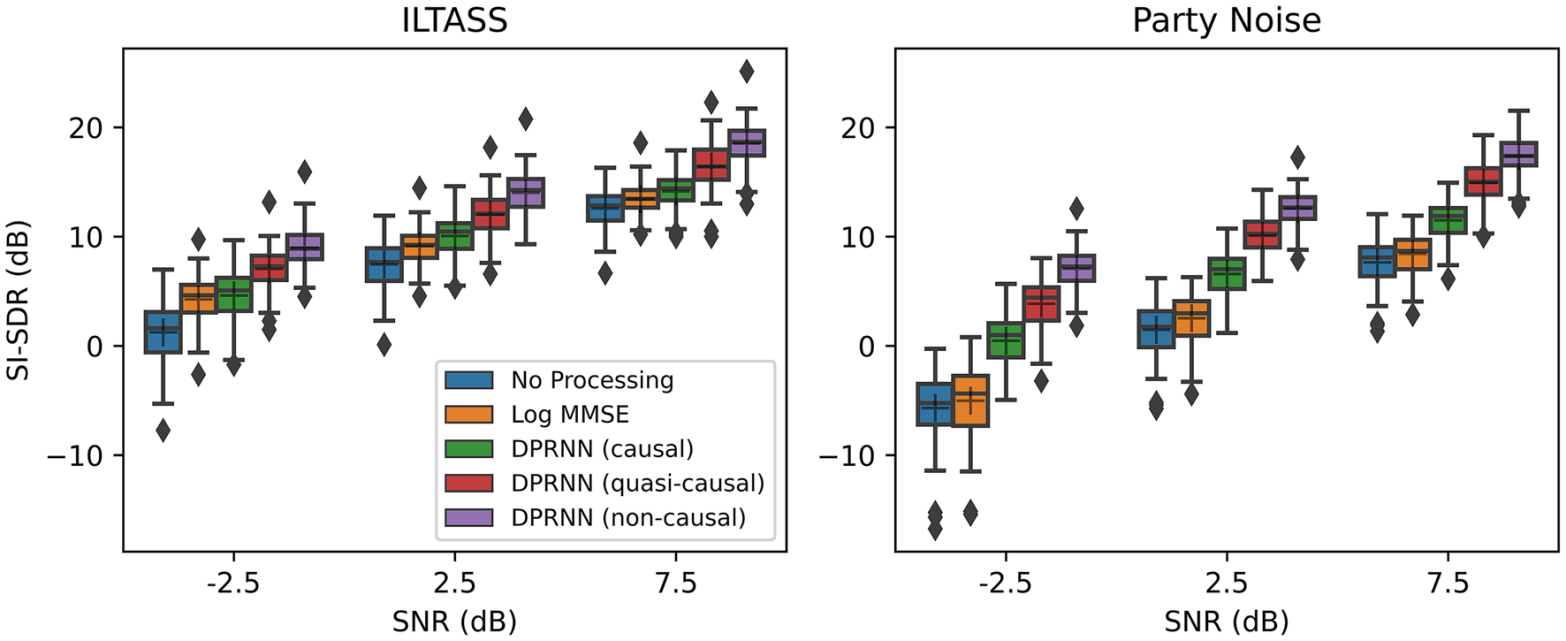
SI-SDRs for the tactile vocoded signal for speech in either the stationary speech-shaped (ILTASS) noise (left panel) or multi-talker party noise (right panel). SI-SDR scores are shown with no noise reduction (blue), with the log-MMSE method (orange), as well as with the causal (green), quasi-causal (red), and non-causal (purple) DPRNN methods. Performance is shown for three SNRs. The mean and median values are illustrated by a cross and a solid line, respectively, while the top and bottom edges of the box show the upper (0.75) and lower (0.25) quartile. Outliers (values of more than 1.5 times the interquartile range) are shown as filled diamonds.

There were two stages to the objective assessment of the quasi-causal DPRNN method. In the first stage, the SI-SDR of the tactile vocoded speech in noise (referenced to time-aligned clean speech) was calculated for the causal, quasi-causal, and non-causal DPRNNs, as well as for the traditional log-MMSE noise reduction method. Performance was assessed for male and female speech either with a stationary noise, which was filtered to match the international long-term average speech spectrum (ILTASS)^34^, or with a multi-talker noise recording from a party. The results for the three SNRs tested are shown in Fig. 1.

A repeated-measures analysis of variance (RM-ANOVA) was performed on the SI-SDRs for the tactile vocoded speech in noise, with the factors: SNR (− 2.5, 2.5, or 7.5 dB), talker (male or female), noise reduction method (log MMSE, causal DPRNN, quasi-causal DPRNN, or non-causal DPRNN), and noise type (stationary or multi-talker). No overall effect of talker was found (*F*(1,59) = 0.7, *p* = 0.400), but there were highly significant effects of SNR (*F*(2,118) = 992.9, *p* < 0.001), noise reduction method (*F*(3,177) = 2077.3, *p* < 0.001), and noise type (*F*(1,59) = 692.4, *p* < 0.001). Significant interactions were found between talker and method (*F*(3,177) = 10.7, *p* < 0.001), talker and noise type (*F*(1,59) = 38.38, *p* < 0.001), method and noise type (*F*(3,177) = 819.0, *p* < 0.001), method and SNR (*F*(6,354) = 48.8, *p* =  < 0.001), and noise type and SNR (*F*(2,118) = 20.4, *p* < 0.001). No interaction was found between talker and SNR (*F*(2,118) = 2.1, *p* = 0.133). Three-way interactions were found between talker, noise reduction method, and noise type (*F*(3,117) = 13.1, *p* < 0.001), between noise reduction method, noise type, and SNR (*F*(6,354) = 216.7, *p* < 0.001), and between talker, noise type, and SNR (*F*(2,118) = 8.7, *p* < 0.001). No significant interaction was found between talker, noise reduction method, and SNR (*F*(6,354) = 1.4, *p* = 0.218). A four-way interaction between all factors was found (*F*(6,354) = 10.9, *p* < 0.001).

Planned *post-hoc t*-tests (corrected for multiple comparisons; see “Methods”) were conducted to assess the effectiveness of each noise reduction method. Statistical tests were only performed for SNRs of 2.5 dB, which were also used in behavioural testing. Compared to no noise reduction, the quasi-causal DPRNN method improved the SI-SDR by 4.6 dB on average for the stationary noise (ranging from 0.8 to 7.1 dB across sentences; standard deviation (SD): 1.4 dB; *t*(59) = 24.7, *p* < 0.001) and by 8.6 dB for the multi-talker noise (ranging from 6.0 to 12.0 dB; SD: 1.3 dB; *t*(59) = 49.9, *p* < 0.001). The causal DPRNN method improved the SI-SDR by 2.6 dB for the stationary noise (ranging from 1.1 to 4.0 dB; SD: 0.7 dB; *t*(59) = 30.1, *p* < 0.001) and by 5.1 dB for the multi-talker noise (ranging from 2.7 to 8.1 dB; SD: 1.0 dB; *t*(59) = 38.2, *p* < 0.001). With the non-causal DPRNN, the SI-SDR was improved by 5.6 dB for the stationary noise (ranging from 2.6 to 10.0 dB; SD: 1.6 dB; *t*(59) = 32.5, *p* < 0.001) and by 11.1 dB for the multi-talker noise (ranging from 8.4 to 14.5 dB; SD: 1.3 dB; *t*(59) = 66.3, *p* < 0.001). Finally, the log-MMSE method improved the SI-SDR by 1.7 dB for the stationary noise (ranging from -0.3 to 3.5 dB; SD: 0.8 dB; *t*(59) = 16.7, *p* < 0.001) and by 1.1 dB for the multi-talker noise (ranging from 0.0 to 2.6 dB; SD: 0.6 dB; *t*(59) = 14.8, *p* < 0.001).

Next, the effectiveness of the quasi-casual DPRNN method was compared between the male and female talkers. A significant difference in SI-SDR was found both with the stationary noise (*t*(59) = 0.8, *p* = 0.417) and multi-talker noise (*t*(59) =  − 4.2, *p* < 0.001). With the stationary noise, the quasi-casual DPRNN improved the SI-SDR by 4.7 dB for the male talker (ranging from 0.0 to 10.4 dB; SD: 2.0 dB) and by 4.4 dB for the female talker (ranging from − 0.4 to 8.8 dB; SD: 2.0 dB). The SI-SDR for the male talker was 0.29 dB better with the stationary noise on average (ranging from − 6.1 to 7.4 dB; SD: 2.8 dB). For the multi-talker noise, the quasi-casual DPRNN improved the SI-SDR by 8.0 dB for the male talker (ranging from 5.0 to 13.1 dB; SD: 1.9 dB) and by 9.2 dB for the female talker (ranging from − 5.7 to 12.2 dB; SD: 1.6 dB). In contrast to the stationary noise, the performance for the female talker with the multi-talker noise was 1.3 dB better on average than the male (ranging from − 5.3 to 6.2 dB; SD: 2.3 dB).

Next, the effectiveness of the quasi-causal DPRNN was compared to the other DPRNN methods and to the traditional log-MMSE method. For the stationary noise, the quasi-causal DPRNN performed better than log-MMSE by 2.9 dB SI-SDR (ranging from 0.4 to 4.8 dB; SD: 1.0 dB; *t*(59) = 22.9, *p* < 0.001) and better than the causal DPRNN by 1.9 dB (ranging from − 0.5 to 4.3 dB; SD: 1.0 dB; *t*(59) = 14.7, *p* < 0.001). The SI-SDR for the non-causal DPRNN was 2.0 dB better than for the quasi-causal DPRNN (ranging from 0.1 to 4.3 dB; SD: 0.8 dB; *t*(59) = 20.0, *p* < 0.001). For the multi-talker noise, the quasi-causal DPRNN performed better than log MMSE by 7.6 dB SI-SDR (ranging from 5.1 to 10.8 dB; SD: 1.3 dB; *t*(59) = 46.4, *p* < 0.001) and better than the causal DPRNN by 3.5 dB SI-SDR (ranging from 1.7 to 5.0 dB; SD: 0.7 dB; *t*(59) = 39.9, *p* < 0.001). The SI-SDR for the non-causal DPRNN was 2.5 dB better than for the quasi-causal DPRNN (ranging from 1.3 to 3.9 dB; SD: 0.6 dB; *t*(59) = 32.9, *p* < 0.001).

Finally, the SI-SDR for the quasi-causal DPRNN was assessed for speech in quiet to establish whether it caused appreciable distortion of speech in quiet. For the male talker, the mean SI-SDR in quiet with the DPRNN was 73.8 dB (SD: 3.2 dB; ranging from 57.5 to 78.2 dB). For the female talker, the mean SI-SDR in quiet was 73.7 dB (SD: 1.9 dB; ranging from 70.2 to 77.8 dB).

**Figure 2:**
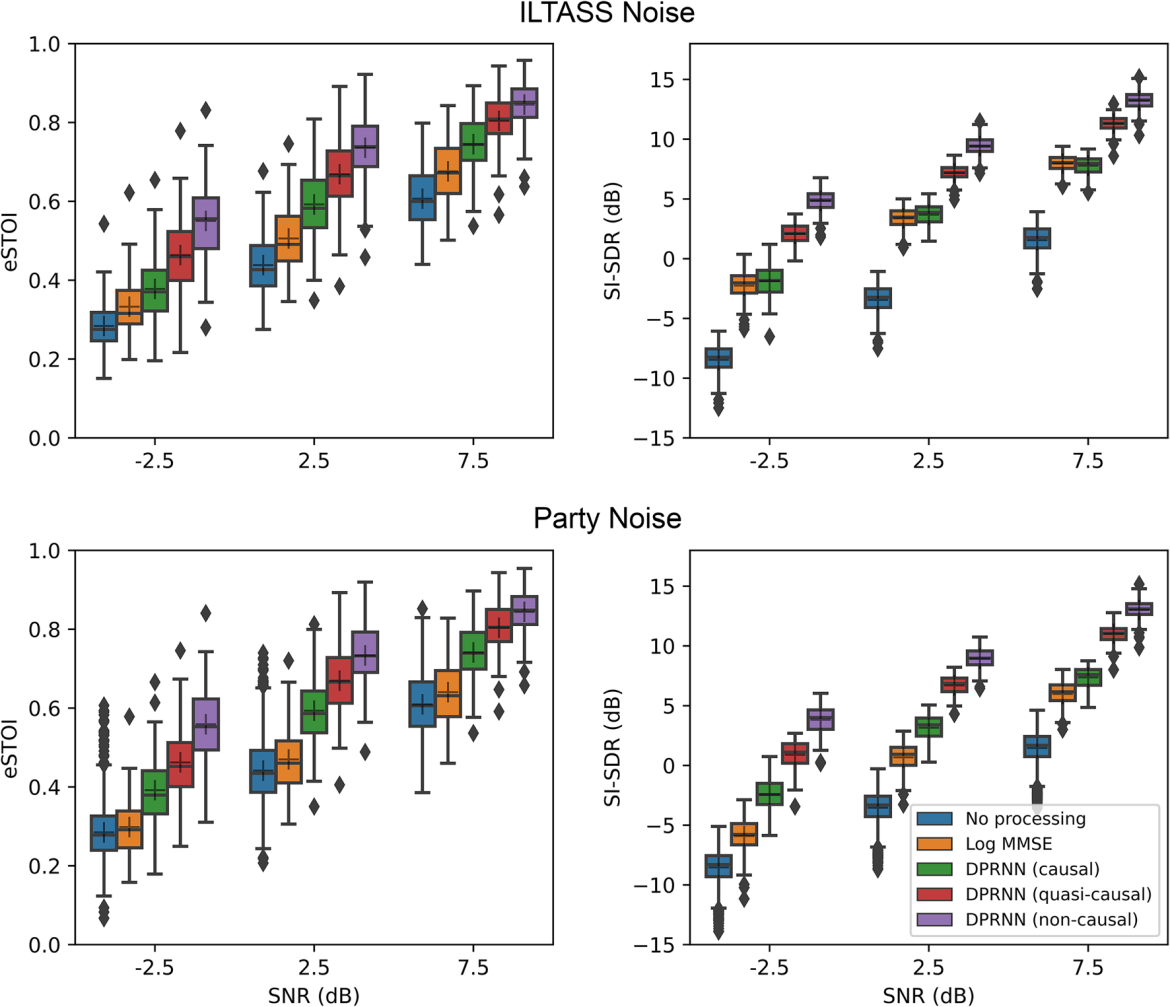
eSTOI scores (left panels) and SI-SDRs (right panels) for the audio signals (without tactile vocoding), with speech in either the stationary ILTASS (upper panels) or multi-talker party (lower panels) noise. SI-SDRs and eSTOI scores are shown with no noise reduction, with the log-MMSE method, and with the three DPRNN methods. As in Fig. 1, the box plots show the median, quartiles, and outliers.

In the second stage of the objective assessment, the effectiveness of each noise reduction method was assessed using the audio signals, without processing through the tactile vocoder. This allows the current quasi-causal DRPNN to be compared more easily with previous studies. Figure 2 shows eSTOI scores and SI-SDRs for speech in noise compared to time-aligned clean speech. At all SNRs, higher average scores were found for the quasi-causal DPRNN compared to no processing. With the stationary noise, SI-SDRs were 10.6, 10.7, and 9.8 dB higher for SNRs of − 2.5, 2.5, and 7.5 dB, respectively, and eSTOI scores were 0.18, 0.23, and 0.19 higher. With the multi-talker noise, SI-SDR scores were 9.4, 10.2, and 9.5 dB higher and eSTOI scores were 0.18, 0.23, and 0.19 higher.

The quasi-causal DPRNN produced higher scores than the traditional log-MMSE method for both the stationary and multi-talker noises. For the stationary noise, SI-SDRs across SNRs were 4.4, 3.8, and 3.3 dB better, respectively, and eSTOI scores were 0.13, 0.16, and 0.12 better. For the multi-talker noise, SI-SDRs across SNRs were 6.8, 6.0, and 5.0 dB better and eSTOI scores were 0.16, 0.20, and 0.16 better. The quasi-causal DPRNN also produced higher scores than the causal DRPNN for both noise types. For the stationary noise, SI-SDRs were 4.0, 3.5, and 3.5 dB higher and eSTOI scores were 0.08, 0.08, and 0.06 higher. For the multi-talker noise, SI-SDRs were 3.3, 3.5, and 3.6 dB higher and eSTOI scores were 0.07, 0.08, and 0.06 higher. Finally, the quasi-casual DPRNN produced lower scores than the non-causal DPRNN for both noise types. For the stationary noise, SI-SDRs were 2.7, 2.2, and 1.9 dB lower and eSTOI scores 0.09, 0.07, and 0.05 lower. For the multi-talker noise, SI-SDRs were 2.9, 2.2, and 2.0 dB lower and eSTOI scores 0.10, 0.06, and 0.04 lower.

### Behavioural assessment

**Figure 3:**
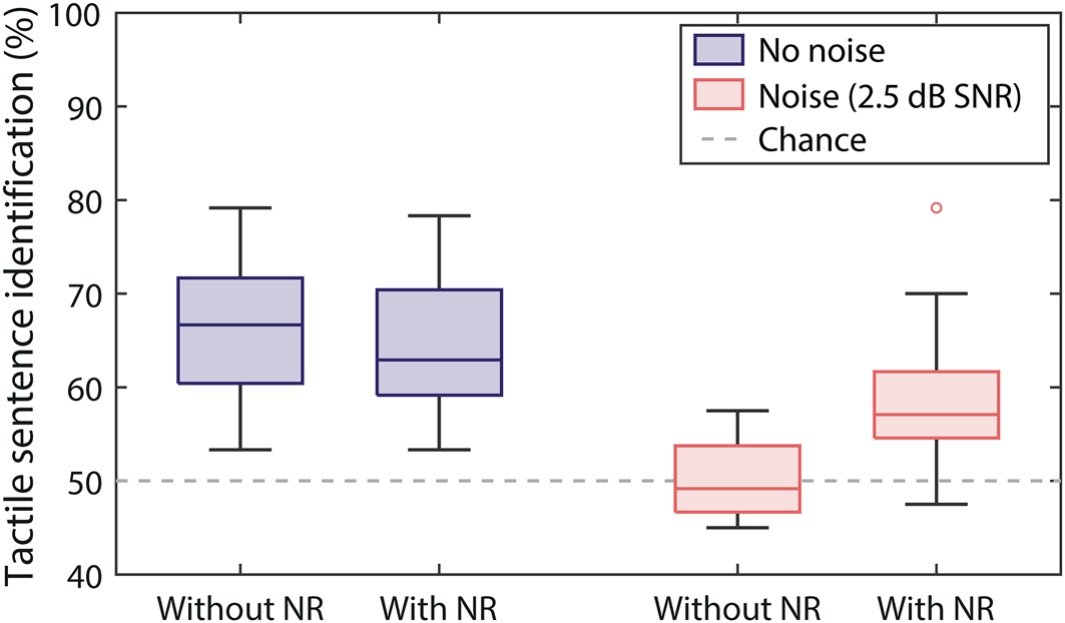
Percentage of sentences correctly identified with and without the quasi-causal DPRNN noise reduction (NR) and with (light red) and without (dark blue) multi-talker (party) background noise at 2.5 dB SNR. Results are shown in box plots, with the horizontal line inside the box showing the median and the top and bottom edges of the box showing the upper (0.75) and lower (0.25) quartile, like in Fig. 1. Outliers (values of more than 1.5 times the interquartile range) are shown as unfilled circles. The whiskers connect the upper and lower quartiles to the maximum and minimum non-outlier value. The dashed grey line shows chance performance.

The percentage of sentences correctly identified in each condition for the 16 participants who took part in the study is shown in Fig. 3. In behavioural testing, the noise reduction was always the quasi-causal DPRNN, and noise was always the multi-talker noise at an SNR of 2.5 dB. The primary analysis of the behavioural data was an RM-ANOVA, with the factors: talker gender (male or female), noise (with or without), and noise reduction (with or without). A significant overall effect of noise (*F*(1,15) = 77.0, *p* < 0.001) and of noise reduction (*F*(1,15) = 7.1, *p* = 0.018) was found, as well as a significant interaction between noise and noise reduction (*F*(1,15) = 15.8, *p* = 0.001). No main effect of talker gender (*F*(1,15) = 1.5, *p* = 0.238) and no interaction between talker gender and any other factor was observed (noise: *F*(1,15) = 1.1, *p* = 0.311; noise reduction: *F*(1,15) = 0.8, *p* = 0.780; noise and noise reduction: *F*(1,15) = 0.1, *p* = 0.711).

Six planned *t*-tests were then run, with multiple comparisons correction applied (see “Methods”). These first established that the noise significantly reduced sentence recognition without noise reduction (*t*(15) = 7.4, *p* < 0.001). Performance reduced by 15.6% (ranging from 25.8 to 0%; standard deviation (SD): 8.5%), from 66.2% in quiet (ranging from 53.3 to 79.2%; SD: 7.9%) to 50.6% in noise (ranging from 45 to 57.5%; SD: 4.2%). Noise reduction improved performance in noise by 8.2% on average (ranging from − 10 to 25%; SD: 7.7%; *t*(15) = 4.3, *p* = 0.003), with performance increasing to 58.8% (ranging from 47.5 to 79.2%; SD: 7.7%). Performance in noise with noise reduction was worse than performance in quiet without noise reduction by 7.5% (ranging from − 3.3 to 16.7%; SD: 5.2%; *t*(15) = 5.7, *p* =  < 0.001). In quiet, no significant change in performance with noise reduction was found (mean reduction in performance of 1.9%; *t*(15) = 1.3, *p* = 0.668).

No difference in the benefit of noise reduction was found across the male and female talkers (*t*(15) = 0.4, *p* > 1). Noise reduction improved performance in noise by 7.5% for the male talker (ranging from − 18.3 to 21.7%; SD: 10.8%) and by 8.9% for the female talker (ranging from − 6.7 to 33.3%; SD: 9.9%). There was also no difference found between the talkers in the effect of noise reduction in quiet (*t*(15) = 0.1, *p* > 1), with average performance worse with noise reduction by 1.8% for the male talker (ranging from a 16.7% reduction to a 10.0% benefit; SD: 8.4%) and by 2.1% for the female talker (ranging from a 15.0% reduction to a 5.0% benefit; SD: 6.3%).

The results can also be broken down into sentence subgroups, consisting of pairs of sentences that are matched for the number of syllables, pairs with the number of syllables differing by one, and pairs with the number of syllables differing by two. For the matched sentences, the mean scores in quiet with and without noise reduction were 59.2% (ranging from 40.0 to 77.5%; SD: 8.7%) and 61.9% (ranging from 37.5 to 75.0%; SD: 10.7%), respectively. In noise, the mean scores with and without noise reduction were 57.3% (ranging from 45.0 to 77.5%; SD: 9.1%) and 50.9% (ranging from 42.5 to 62.5%; SD: 7.4%), respectively. For sentences with the number of syllables differing by one, the mean scores in quiet with and without noise reduction were 67.7% (ranging from 57.5 to 82.5%; SD: 7.5%) and 70.0% (ranging from 55.0 to 80.0%; SD: 7.4%), respectively, and, in noise, were 59.7% (ranging from 37.5 to 75.0%; SD: 10.4%) and 46.7% (ranging from 40.0 to 57.5%; SD: 4.7%). For sentences with the number of syllables differing by two, the mean scores in quiet with and without noise reduction were 65.9% (ranging from 45.0 to 82.5%; SD: 11.7%) and 66.7% (ranging from 50.0 to 92.5%; SD: 11.8%), and, in noise, were 59.2% (ranging from 40.0 to 85.0%; SD: 11.9%) and 54.1% (ranging from 45.0 to 67.5%; SD: 7.0%).

Finally, exploratory *post-hoc* correlation analyses were run (see “Methods”). These assessed the relationship between either participant age or vibro-tactile detection thresholds and either sentence identification scores in quiet without noise reduction or the improvement in sentence identification scores in noise with noise reduction. No evidence of a correlation between vibro-tactile detection thresholds and sentence identification was found (in quiet: *r* = 0.13, *p* = 0.632; benefit of noise reduction: *r* = 0.10, *p* = 0.713). There was also no clear evidence of a correlation between age and sentence identification in quiet (*r* = 0.37, *p* = 0.161). There was a trend for older participants to get more benefit of noise reduction, but this correlation between age and benefit did not reach significance (*r* = 0.47, *p* = 0.067).

## Discussion

The objective assessment for tactile speech-in-noise showed that the quasi-causal DPRNN noise reduction method substantially outperforms traditional noise reduction methods, both for stationary and multi-talker background noises. As expected, the largest gains were found for multi-talker background noise (7.6 dB SI-SDR compared to 2.9 dB SI-SDR at an SNR of 2.5 dB), where traditional methods are known to particularly struggle. Behavioural testing confirmed that the effectiveness of the quasi-causal DPRNN shown in the objective assessment translates to a substantial improvement in tactile sentence identification, without impairing performance for speech in quiet. The quasi-causal DPRNN improved behavioural speech-in-noise scores by 8.2%, which amounted to a recovery of around half of the performance that was lost when noise was added. The acoustic characteristics of the multi-talker noise and the SNR of 2.5 dB are representative of scenarios in which people with hearing loss commonly struggle in their everyday lives. The improved noise-robustness with the real-time-feasible quasi-causal DPRNN could dramatically increase the utility of haptic hearing aids, both when used as stand-alone sensory substitution devices and when used for sensory augmentation with CIs and other hearing-assistive devices.

A small difference in the performance of the quasi-causal DPRNN between the male and female talkers was observed in objective testing. However, this did not translate to a measurable difference in behavioural performance. The talkers used for testing were not included in the training set and varied substantially in their acoustic properties (see “Methods”). The small objective difference in performance between talkers can likely be reduced by using a training corpus with a more diverse set of talkers^35^. However, the finding of only small objective differences, and no behavioural difference, suggests the current quasi-causal DPRNN would generalise well to other talkers.

The causal, quasi-causal, and non-causal DPRNN methods all markedly improved both SI-SDR and eSTOI scores in the audio domain, for both noises tested. As expected, better performance was observed when more future information was included, with performance increasing between causal and quasi-causal processing and between quasi-causal and non-causal processing. The traditional log-MMSE method produced the smallest improvement in objective scores, with negligible improvements for the multi-talker (party) noise. It is therefore thought unlikely that log-MMSE would have yielded any improvement in behavioural tactile speech-in-noise performance. The current results are in line with previous studies showing that the DPRNN and other deep-learning-based noise reduction methods can improve objective speech intelligibility outcomes in both non-stationary and stationary noise^17,18,21^. In contrast, traditional noise reduction methods fail to achieve significant improvements in non-stationary conditions and only provide small benefits in stationary noise^16^. The average improvements in eSTOI scores of around 0.2 with the DPRNN methods was comparable or better than found previously^36^, although direct comparisons of the objective scores to previous work are difficult because different stimuli were used for evaluation.

The objective results differed somewhat for the tactile domain compared to the audio domain. While consistent benefits in SI-SDR scores for the multi-talker noise were observed in the audio domain for log MMSE, these were largely absent in the tactile domain. For the DPRNN methods, the pattern of SI-SDR improvements was consistent between audio and tactile domains. However, in the tactile domain, SI-SDR results were compressed into a smaller overall range, reducing any differences between methods. This may be explained by tactile vocoding lessening the differences between signals, by reducing them to only eight frequency bands and applying low-pass filtering. Further work is required to determine whether SI-SDR is an effective predictor of tactile speech recognition in noise. Further work might also assess whether objective and behavioural equivalents of speech quality (e.g., the perceptual of evaluation of speech quality (PESQ) metric^37^), can also be established for tactile speech.

**Figure 4:**
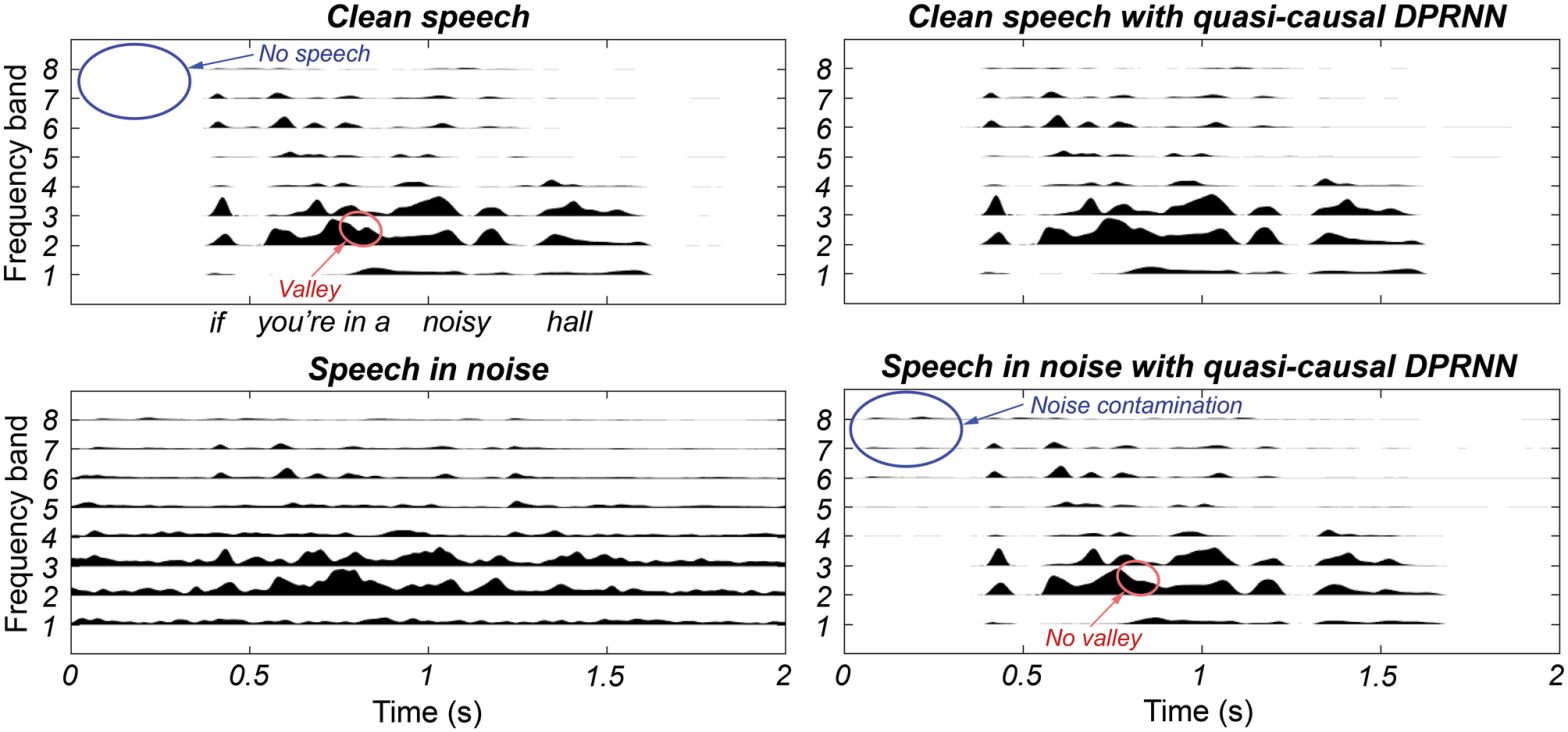
Amplitude envelopes extracted from each of the eight frequency bands for the audio-to-tactile vocoder method used in objective and behavioural testing. Band 1 represents the lowest frequency band and band 8 represents the highest frequency band. Clean speech (top panels) and speech in noise (bottom panels) are shown both with (right panels) and without (left panels) the quasi-causal DPRNN. The speech is the sentence, “if you’re in a noisy hall”, spoken by the female talker used for testing in the current experiment. This was selected to be a representative example of both the successes and failures of the quasi-causal DPRNN. The noise was the multi-talker party noise used in the current experiment. The blue circles highlight an example of noise contamination after processing with the quasi-causal DPRNN, and the red circles highlight smearing of the amplitude envelope. Envelope amplitudes are in linear units and are normalised to the maximum amplitude in each panel.

While behavioural performance in background noise was substantially improved by the quasi-causal DPRNN, it was unable to fully recover performance to match that in quiet. There are several possible reasons for this. Figure 4 shows the amplitude envelopes from the tactile vocoder used in the current experiment for one of the sentences tested. Envelopes are shown with and without the multi-talker background noise and both with and without the quasi-causal DPRNN. By comparing the clean speech without the DPRNN (top left panel) to the speech in noise with the DPRNN (bottom right panel), it can be observed that, while strong noise rejection was achieved, some artefacts remained (for example, before the start of the sentence in channel 8, which is highlighted with blue circles). These artefacts may have acted as distractors that increased temporal uncertainty^38–40^ and disrupted estimation of key speech landmarks, such as sentence, syllable, and phoneme start and end points. Another subtler difference between the clean envelopes and the envelopes with the DPRNN is the smoothing of some envelope modulations. An example can be seen in channel 2 for the words “…in a…” (highlighted with red circles). This smoothing might reduce the salience of speech segments, particularly for speech tokens in which rapid envelope changes signal phoneme identity (plosive consonants) or aid speech segmentation.

Noise reduction methods balance removal of background noise and distortion of the speech signal^41^. For practical applications, speech distortions can be mitigated by limiting the aggressiveness of the noise-reduction processing, for example by mixing the DPRNN output signal with the original speech-in-noise signal. The optimal balance usually depends on several factors that differ between users, including the acoustic challenges typically faced (e.g., the SNR and the character of the background noise) and the amount of speech distortion that the user can tolerate. Data on user characteristics is currently lacking for haptic hearing aids, and further work is required to establish the optimal balance of noise rejection and speech distortion.

Some of the factors that impaired performance in noise may have had reduced impact or been overcome entirely if participants had received training with tactile speech. Previous studies both with tactile sensory substitution for speech in quiet^11^ and sensory augmentation for speech in noise^3,4,7^ have shown that training significantly improves performance. This improved ability to extract speech information from tactile stimulation might improve robustness to noise artefacts. This suggestion is supported by hearing studies, which have shown that training can reduce the impact of masking sounds^42,43^. Future work should establish the importance of training when assessing noise reduction methods for tactile speech, particularly in allowing users to reject artefacts.

Experiential factors other than direct tactile speech training might also be important predictors of tactile speech performance. For example, indirect training to extract auditory information from vibration, when using a tool or a musical instrument, might generalise to tactile speech. A previous study highlighted the case of a participant who, before they received their CI, had learned to play the flute by feeling its vibrations^3^. It was speculated that this may have contributed to the participant’s unusually large improvement in speech-in-noise performance with haptic stimulation even before receiving tactile speech training. Further investigation is required to establish whether such factors are predictive of tactile speech performance.

A major challenge for noise reduction methods is the selection of the appropriate talker in a multi-talker scene, particularly when the target and a competing talker have a similar intensity. In the current study, competing speech was well below the level of the target speech, meaning there was little ambiguity as to which talker should be targeted by the DPRNN. One method for improving the ability of hearing aids to target the talker of interest in challenging multi-talker scenarios is directional filtering through beamforming, which typically assumes that the user is facing the sound source of interest. Recently, more advanced methods have been used to determine the sound source of interest, such as eye gaze tracking, though so far with limited success (e.g.,^44^). Further work is required to characterise the performance of the quasi-causal DPRNN under conditions with high target-talker ambiguity, and the effects of methods, such as beamforming or target speech extraction^45^, on DPRNN performance.

The quasi-causal DPRNN might be further optimized for tactile speech to improve noise robustness and reduce computational complexity. One optimisation might be to place the DPRNN later in the tactile signal-processing chain. In the current study, the DPRNN was used as front-end noise reduction, being applied to the audio before the tactile vocoder. Future studies could explore whether, for example, the DPRNN is more effective and efficient if processing only the audio from the eight tactile vocoder audio filter bands, which are designed to focus on frequencies most important to speech perception. Alternatively, the DPRNN could be trained specifically to extract the clean tactile speech envelopes from noise, rather than the clean audio. This would allow the speech synthesis (decoder) stage of the DPRNN algorithm to be removed and therefore for the computational complexity and processing latency to be reduced. Furthermore, the much smaller spectral resolution of the tactile signals (eight channels in the current study) compared to the spectral resolution of acoustic audio may allow a substantial reduction in the complexity of the other DPRNN stages, without compromising performance. Finally, power efficiency might be optimised by selectively activating the DPRNN only when background noise is detected. There are several existing environmental classification algorithms that could be used to achieve this (e.g.,^33^).

Another approach to improving the effectiveness of the DPRNN might be to provide it with multiple microphone signals, rather than single-channel audio. In previous work, the audio received at microphones behind each ear was converted to haptic stimulation on each wrist so that spatial hearing cues could be exploited through haptics. This has been shown to improve sound localisation^46–49^ and speech recognition for spatially-separated speech and noise in CI users^9^. The improvements in speech-in-noise performance were likely achieved, at least in part, through access to better speech information from the ear with the best SNR. In future iterations of the DPRNN, this “better-ear-listening”, or more advanced multi-microphone noise-reduction methods (e.g.,^50,51^) could be exploited.

There are important limitations to the current study that should be noted. Firstly, a new tactile sentence identification task was used, with a forced-choice method deployed to avoid floor effects and circumvent the need for prolonged tactile speech training. Unlike some previous methods for assessing tactile speech performance, which focus on phonemes^10,14^ or words^11^, this method was intended to allow the use of a range of key speech processing factors. These include segmentation and recognition of phonemes and words in running speech and integration of sensory and higher-level (e.g., top-down) processing. Improved ability to discern sentences in background noise is expected to strongly predict whether haptic stimulation can work effectively, either when delivered alone or when supplementing lip reading or a degraded acoustic signal (e.g., through a CI). However, further work is required to conclusively establish this.

Another limitation was that participants in the current study had no known hearing loss, unlike the target user group for haptic hearing aids, which consists primarily of CI users and those with a profound hearing loss who can’t access CI technology. Previous studies have found no difference in tactile speech performance in quiet^10,12,14,52^ or in background noise^3,4,9^ between those with and without hearing loss. However, there is evidence that tactile sensitivity might be higher in those with congenital deafness^53^. This might mean that the current results underestimate performance for some groups of people with hearing loss.

The participant group also did not cover the full age range of the target user group. Previous studies have found no relationship between age and tactile speech performance^3,4,9,10,14^ and, in the current study, there was also no evidence of a correlation between age (which spanned 19 years) and tactile speech performance in quiet. The current study found weak evidence that older users might perform better when extracting speech from background noise, but this result needs to be replicated in a larger study to become compelling. While previous work has found no worsening with age of either temporal gap detection^54^ or intensity discrimination^48,55^ for vibro-tactile tones, detection thresholds are known to worsen with age^56^. This may mean that adjusting the dynamic range of tactile stimulation to take account of differences in sensitivity will be important for maximizing the effectiveness of haptic hearing aids.

In addition to age, demographic factors such as the duration of deafness might be important predictors of tactile speech performance (see Fletcher^57^ and Fletcher and Verschuur^5^ for extended discussion). The success of haptic hearing aids is likely to be heavily determined by the ability of users to integrate tactile speech information with speech information gathered through lip reading and through any acoustic or electrical hearing. There is evidence that this integration differs across groups. For example, CI users who are implanted after several years of deafness have been found to integrate audio and visual information less effectively than those who are implanted early^58–60^. This may mean that those implanted early will benefit more from haptic hearing aids. Another important factor might be the visual acuity of the user. Vision is thought to calibrate auditory responses^61^ and to guide auditory perceptual learning^62^, and could play a similar role for tactile speech. Further work is required to establish the groups that can benefit most from haptic hearing aids.

The current study showed that a real-time-feasible quasi-causal DPRNN noise reduction method can substantially improve tactile sentence identification in noise, while not impairing performance in quiet. The greater design space available for haptic hearing aids (and therefore capacity to use larger, more powerful microprocessors) compared to other hearing-assistive devices makes deployment of advanced noise-reduction methods, such as the DPRNN, more viable. The quasi-causal DPRNN could dramatically increase the effectiveness of haptic hearing aids both when used for sensory augmentation alongside devices such as CIs and when used to aid the many millions of people around the world who cannot access CI technology.

## Methods

### Participants

Table 1 shows the characteristics of the 16 adults who completed the behavioural experiment. There were 6 males and 10 females, with an average age of 24 years (ranging from 18 to 37 years). Participants all had normal touch perception, which was assessed through a screening questionnaire and by measuring vibro-tactile detection thresholds at the fingertip (see “Procedure”). All participants had British English as their first language and reported no hearing problems. Participants were each paid an inconvenience allowance of £20 for taking part.

**Table 1:** Participant characteristics.

ID	31.5 Hz thresh (m/s^−2^)	125 Hz thresh (m/s^−2^)	Wrist temp. (°C)	Wrist height/width (mm)	Wrist circum (mm)	Dom. hand (L/R)	Age (yrs.)	Sex (M/F)
1	0.02	0.08	30.3	39/58	166	R	36	M
2	0.02	0.09	28.8	36/48	150	R	22	F
3	0.04	0.08	31.6	36/48	149	L	18	F
4	0.05	0.07	31.6	41/52	160	R	29	M
5	0.06	0.41	32.2	43/55	172	R	37	M
6	0.03	0.03	28.4	32/49	149	R	21	F
7	0.05	0.03	25.6	44/59	171	R	19	M
8	0.05	0.10	31.8	41/50	160	R	23	M
9	0.05	0.06	28.4	43/51	168	R	18	F
10	0.08	0.15	30.1	41/48	148	L	20	F
11	0.08	0.20	28.6	40/49	154	R	20	F
12	0.02	0.03	31	35/51	148	R	28	F
13	0.01	0.05	31	41/51	166	R	26	F
14	0.09	0.29	27.9	39/51	162	R	20	M
15	0.05	0.05	31.2	38/55	153	R	18	F
16	0.02	0.06	34	33/46	138	R	21	F
Mean	0.05	0.11	30.2	39/51	157	–	24	–

Each participant’s vibro-tactile detection thresholds (measured during screening), wrist temperature at the start of testing, wrist dimensions, dominant hand, age, and sex are shown.

### Stimuli

#### Test material

The tactile stimulus in the experiment phase (after screening), was generated using the EHS Research Group Sentence Corpus. This contained 83 sentences, each spoken by both a British English male and British English female talker. The sentences were taken from readings (connected discourse) of a public engagement article written in a semi-conservational style^63^. They contained a range of natural variations in prosodic pattern, speaking rate, pitch, and phoneme pronunciation.

**Figure 5:**
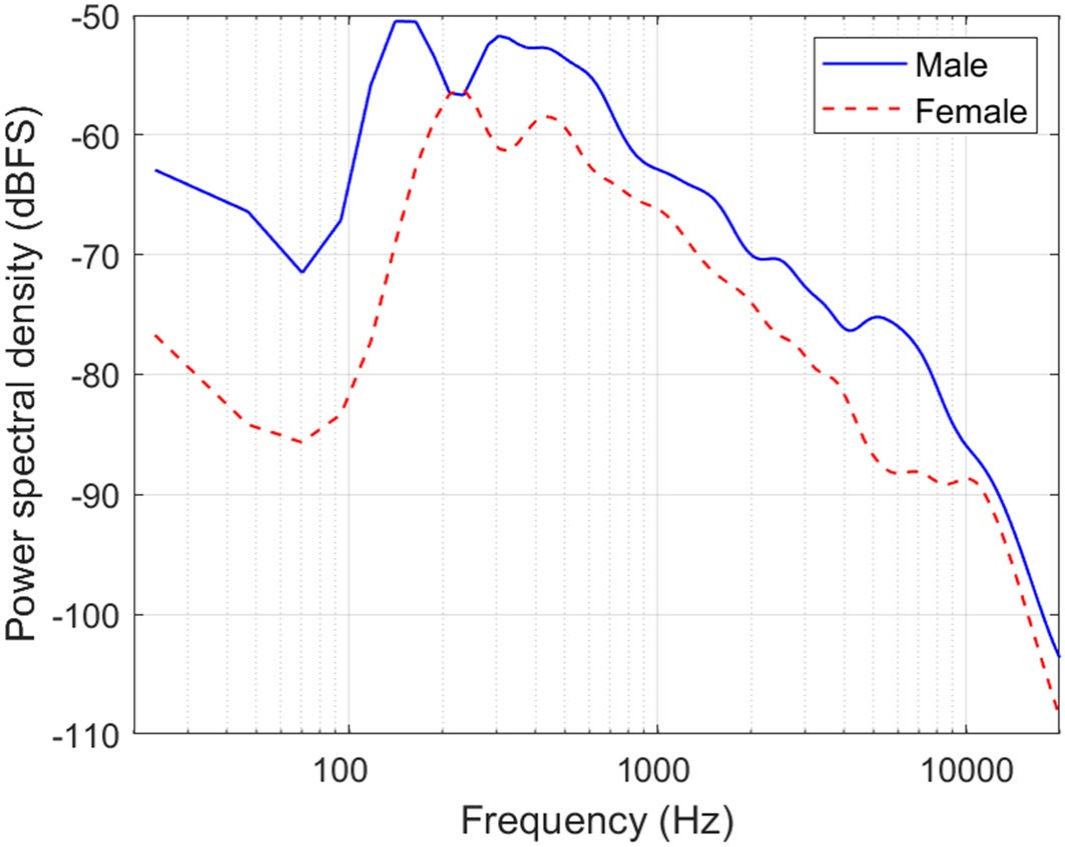
The long-term average spectrum of the male and female talker from the EHS Research Group Sentence Corpus, with no normalisation applied. The spectrum was calculated from the average power spectral density (Hann windowed, with a 96 kHz sample rate, an FFT length of 4096, and a hop size of 2048). The average power spectral density was Gaussian-smoothed with a 1/3 octave resolution.

The long-term spectrum for each talker is shown in Fig. 5. The male talker had an average fundamental frequency of 147.9 Hz (ranging from 80.5 to 220.7 Hz; SD: 19.0 Hz) and the female talker had an average fundamental frequency of 205.5 Hz (ranging from 108.2 to 285.7 Hz; SD: 31.4 Hz). The fundamental frequency (estimated using a Normalized Correlation Function) and the harmonic ratio (the ratio of the energy at the fundamental frequency to the total energy) were determined using the MATLAB “audioFeatureExtractor” object (MATLAB R2022b). A 300-ms Hamming window was used, with a 30-ms overlap length. Samples were included in the analysis if their harmonic ratio was greater than 0.8.

The EHS Research Group Sentence Corpus was recorded in an anechoic chamber at the Institute of Sound and Vibration Research (UK). The audio was recorded using a Rode M5 microphone and RME Fireface UC soundcard (with a 96 kHz sample rate and a bit depth of 24 bits). The microphone was 0.2 m from the talker’s mouth.

A subset of 60 sentences from the EHS Research Group Sentence Corpus were used in the behavioural and objective assessments (see “Table 2”). Each of the 60 sentences were spoken by both the male and female talker so that there were 120 speech samples in total. The cross section of sentences had a range of total durations and contained a variety of prosodic patterns with different pitch contours, phoneme inventories, syllable numbers, and rates. The sentences were grouped in pairs, with 10 of the pairs having the same number of syllables, 10 of the pairs differing by 1 syllable, and 10 of the pairs differing by 2 syllables. The pairs were intended to span a wide range of difficulties determined by a range of factors. For example, the sentences “They keep on getting better and better” and “Together with a team of researchers” do not differ in syllable count, have a similarly high proportion of obstruent phonemes, and have a very similar total duration (durations differ by 100 ms or less). In contrast, the sentences “That people can wear outside the lab” and “This is one of the main ways your brain works out”, have a different number of syllables, a different number of nasal sounds, and a relatively large difference in total duration (a 600-ms difference for the female talker and a 400-ms difference for the male talker).

**Table 2:** The subset of 30 sentence pairs used in the behavioural experiment.

Syllable difference	Number of syllables	Sentence 1 text	Sentence 2 text
Matched	3	However	Did it work?
Matched	4	Blocked by your head	For example
Matched	5	Over and over	And the one they chose
Matched	5	For many people	Interestingly
Matched	6	It tells you where things are	But to reach your left ear
Matched	8	In India, for example	When you block it by closing a door
Matched	8	If you're in a noisy hall	This can cause a lot of problems
Matched	9	We were very pleased with what we found	They could help people across the world
Matched	10	They keep on getting better and better	Together with a team of researchers
Matched	13	We calculated how well they located the sound	And the music blaring from the speaker to your left
1 Diff	2/3	So far	Who is shy
1 Diff	2/3	Quieter	We hoped that
1 Diff	4/5	To get a job	Through another sense
1 Diff	4/5	Ears, nose, and mouth	To make this happen
1 Diff	6/7	And who likes to show off	Between the correct speaker
1 Diff	6/7	Touch and temperature	When using only their ears
1 Diff	11/12	You listen all the more closely for footsteps	This difficulty isn't a temporary one
1 Diff	11/12	And is always hungrily searching for more	Perhaps we can send the missing information
1 Diff	14/15	They might be especially useful in poorer countries	They tended to be much closer to the correct location
1 Diff	14/15	Which are a type of surgically fitted hearing aid	Less than a third of children with hearing problems go to school
2 Diff	7/9	Our volunteers performed best	If you don't know where it's coming from
2 Diff	7/9	Could overcome these problems	You can see this from the big blue bar
2 Diff	7/9	As they go about their day	As illustrated in figure one
2 Diff	7/9	That we took advantage of	The wristbands we are developing
2 Diff	9/11	We measured this distance in degrees	Like the voice of the person in front of you
2 Diff	9/11	That people can wear outside the lab	This is one of the main ways your brain works out
2 Diff	10/12	Or expensive medical equipment	Adults with hearing problems in poorer countries
2 Diff	10/12	Have been invented to solve this problem	And so are often forced to live in poverty
2 Diff	11/13	But because a sense isn't working properly	If you're trying to follow a conversation
2 Diff	11/13	Now we're looking to create a device	Were converted into vibration on the right wrist

The 60 sentences listed were also used in the objective assessment (but pairings were disregarded). For each pair, the table shows the difference in syllable count between the sentences (matched, one different, or two different), the total syllable count for each sentence, and the sentence text. The sentence text is the same for the male and female talkers.

In behavioural testing, the conditions with background noise used a non-stationary multi-talker recording from a party that was made by the Australian National Acoustic Laboratory^64^. The noise sample has a long-term spectrum that matches the international long-term average speech spectrum (ILTASS)^34^. This noise was selected to reproduce real-world challenges that haptic hearing aid users would face, including the typical fluctuations and modulations due to background speech and other non-stationary sounds. In objective testing, an additional stationary noise was used, which was filtered so that its long-term spectrum matched the ILTASS. The stationary ILTASS noise served as a comparison condition, with maximal energetic masking, that is widely used in speech perception studies. It should be noted that the DPRNN was exclusively trained with recordings of realistic background noises and not with similar artificially generated noises.

For the behavioural speech-in-noise conditions, the speech and noise signals were mixed with a 2.5 dB SNR. In the objective assessment, SNRs of − 2.5 and 7.5 dB were additionally tested. Importantly, the RMS level of the speech was calculated with silences removed (though note that silences were not removed from the stimulus for presentation). Silent sections were identified by extracting the speech amplitude envelope using a Hilbert transform and a zero-phase 6th-order Butterworth low-pass filter, with a cut-off frequency of 23 Hz. Sections of the speech where the amplitude envelope dropped below 10% of the maximum were removed for the RMS level calculation. This meant that SNR setting in the current study was lower than for comparable studies where the silences were not removed for the SNR calculation. For the EHS Research Group Sentence Corpus, the 2.5 dB SNR was 2.6 dB lower on average when silences were removed than when they were not (ranging across sentences from 0.9 to 4.7 dB lower; SD: 0.7 dB). For the more naturalistic speech material used in the current study—which contains a variety of prosodic characteristics, with differing syllabic stress patterns, speaking rate and overall modulation characteristics – silence-stripping was deemed important to achieving a stable SNR across sentences.

On each trial, the masker duration was set to have a randomly selected gap of between 500 and 1500 ms before and after the longest speech stimulus of the sentence pair. This was done to exclude the possibility that participants could learn when the sentence onset or offset would be and use total duration cues without detecting the speech signal. If the speech was the shorter sentence of the pair, it was located within the masker at a random point within the time window for the longer sentence. The speech and noise samples were ramped on and off with a 50-ms raised-cosine ramp. A new randomly selected section of the full noise sample was used in each trial. The audio generated for behavioural testing was also used in the objective assessment.

#### Noise-reduction methods

In both behavioural and objective testing, the audio was first downsampled to 16 kHz for conversion from audio to haptic stimulation. This matches the typical sample rate available in compact wearable audio devices, such as CIs and hearing aids. In the behavioural assessment, only the quasi-causal DPRNN noise-reduction method was tested. For conditions with the quasi-causal DPRNN applied, the audio was first processed with the DPRNN algorithm, before being converted to tactile stimulation using the tactile vocoder (see following section).

**Figure 6:**
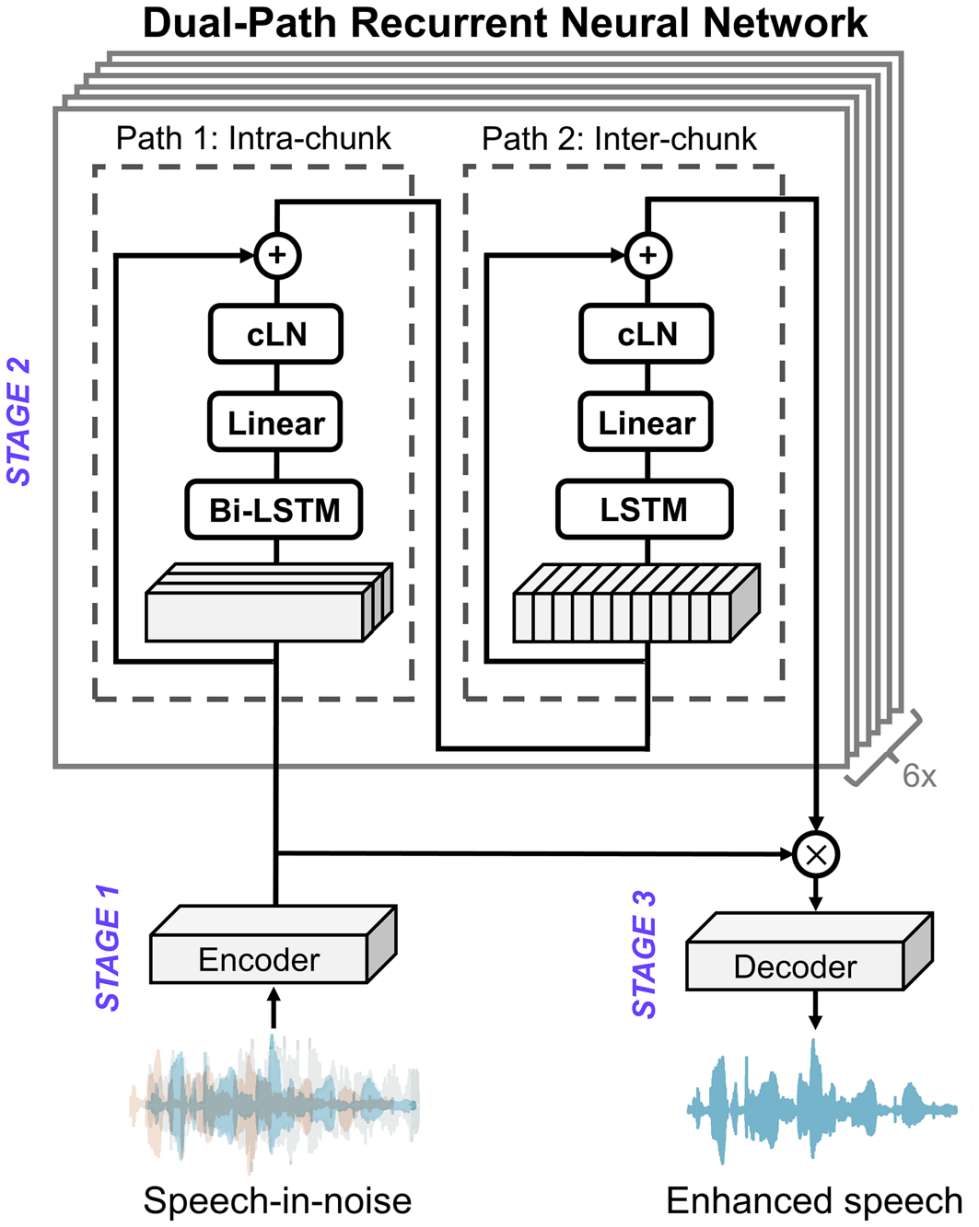
Schematic representation of the quasi-causal dual-path recurrent neural network (DPRNN), with layer descriptors: LSTM for long-short term memory, Bi-LSTM for bidirectional LSTM and cLN for channel-wise layer normalization.

The DPRNN consisted of an end-to-end time-domain Audio Separation Network (TasNet) with three stages (see Fig. 6). In the first stage, a learned encoder block transformed the time-domain audio frames into a 2D feature space (similar to a time–frequency representation). The next stage consisted of a masking network, which processed consecutive chunks within the latent feature space to estimate a noise-reduction mask. This mask was then applied to the noisy input speech representation to remove the background noise and to produce the enhanced speech signal. The final stage consisted of a learned decoder block, which transformed the enhanced speech signal back into a time-domain audio output signal.

For the encoder and decoder blocks, the DPRNN algorithm used 1D-convolutional layers. The 1-D convolution layer (CONV1D) transformed a single-channel audio waveform (1-D) into 128 channels. This was achieved by individually convolving 128 filter kernels with the input waveform, using a kernel size of 16 (1 ms) and a stride of 4 (0.25 ms) samples^22^. The masking network consisted of 6 blocks of the DPRNN. The DPRNN has two paths, one for processing information in the current chunk only, and one for processing information across chunks. The intra-chunk processing path used bi-directional LSTM layers, whereas the inter-chunk path used uni-directional LSTM layers.

The DPRNN algorithm was implemented using the publicly available Asteroid PyTorch toolkit^65^. The model was retrained for use in the current study with a large dataset of speech-in-noise stimuli comprising of speech utterances from the LibriSpeech corpus^66^ and noise samples from the WHAM! stimulus set^67^ at various SNRs (total of 360 h). The SNRs were sampled from a uniform distribution between − 6 and 10 dB, with 5% of the speech samples retained to ensure that performance for clean speech had not degraded. These speech and noise stimuli differed from those used during behavioural and objective testing of the DPRNN. The training made use of the Adam optimizer^68^ and the scale-invariant signal-to-distortion ratio^69^ as loss functions for a total of 200 epochs. The training of the DPRNN algorithm was performed on three NVIDIA A100 40GB Tensor Core graphics processing units.

In the objective assessment, alternative causal and non-causal DPRNN noise-reduction methods were tested in addition to the quasi-causal DPRNN, with different parameters and different use of future information. For the non-causal DPRNN, all LSTM units were bi-directional. The bottleneck and hidden dimensions were set to 128 units and global normalization layers were used. For the quasi-causal DPRNN, the chunks processed by the masking network consisted of 20 frames, which limited the use of future information to 5.75 ms to allow real-time processing to be feasible. The causal DPRNN used no future information. For both the quasi-causal and causal DPRNN, bottleneck and hidden dimensions were set to 256 units and channel-wise normalisation was used to ensure causal processing of information from the current chunk only. This configuration ensured that the DPRNN models had approximately the same number of parameters, allowing a fair comparison between methods. All models were trained on the same data and used the same training regime.

In addition to the DPRNN, the log-MMSE noise-reduction method was assessed^29^. The log-MMSE method is based on statistical modelling and is one of the most popular noise reduction methods for hearing-assistive devices. It was selected to represent traditional noise-reduction methods in the current study as it has been found to be among the best performing traditional approaches^30^. The audio was processed with the log-MMSE method using the MATLAB (2022b) implementation from Loizou^16^.

#### Tactile vocoder and vibro-tactile stimulation

Following downsampling to 16 kHz and, for some conditions, the application of noise reduction, the signal was converted to vibro-tactile stimulation using a tactile vocoder (following the method used previously by Fletcher, et al.^10^). The audio was first passed through a 512th-order FIR filter bank with eight frequency bands, which were equally spaced on the auditory equivalent rectangular bandwidth scale^70^ between 50 and 7000 Hz. Next, the amplitude envelope was extracted for each frequency band using a Hilbert transform and a zero-phase 6th order Butterworth low-pass filter, with a cut-off frequency of 23 Hz (targeting the envelope modulation frequencies most important for speech recognition^71,72^). These amplitude envelopes were then used to modulate the amplitudes of eight fixed-phase vibro-tactile tonal carriers.

The eight tactile tones had frequencies of 94.5, 116.5, 141.5, 170, 202.5, 239, 280.5 and 327.5 Hz. These remained within the frequency range that can be reproduced by the latest compact haptic actuators and were spaced so as to be discriminable based on vibro-tactile frequency discrimination thresholds from the dorsal forearm^73^. A different gain was applied to each tone to make them equally detectable across frequency, based on previously measured tactile detection thresholds^10,74^. The gains were 13.8, 12.1, 9.9, 6.4, 1.6, 0, 1.7, and 4 dB, respectively. Tactile stimuli were scaled to have an equal RMS amplitude, with a nominal level of 1.2 G (141.5 dB ref. 10^−6^ m/s^2^). This intensity can be produced by a range of compact, low-powered haptic actuators that are suitable for a wearable device.

Throughout behavioural speech identification testing, pink noise was presented through headphones at a level of 60 dBA to ensure any auditory cues were masked. During familiarisation, there was no masking noise, and the speech audio was played through the headphones at 65 dBA.

### Apparatus

During behavioural testing, participants sat in a vibration isolated, temperature-controlled room (mean temperature: 23 °C; SD: 0.45 °C). The room temperature and participant’s skin temperature were measured using a Digitron 2022T type K thermocouple thermometer, which was calibrated following ISO 806012-56:2017^75^ using the method described in Fletcher, et al.^10^.

In screening, vibrotactile detection thresholds were measured with a HVLab Vibro-tactile Perception Meter^76^. The circular probe was 6 mm in diameter and contacted the skin through a circular hole in a rigid surround that had a 10 mm diameter. The probe gave a constant upward force of 1N. The downward force applied by the participant was measured using a force sensor built into the surround. This sensor was calibrated using Adam Equipment OIML calibration weights and the amount of force being applied was displayed to the participant. The Vibro-tactile Perception Meter output was calibrated using its built-in accelerometers (Quartz Shear ICP, model number: 353B43) and a Brüel & Kjær (B&K) Type 4294 calibration exciter. The system conformed to ISO-13091-1:2001^77^ and the stimuli had a total harmonic distortion of less than 0.1%.

**Figure 7:**
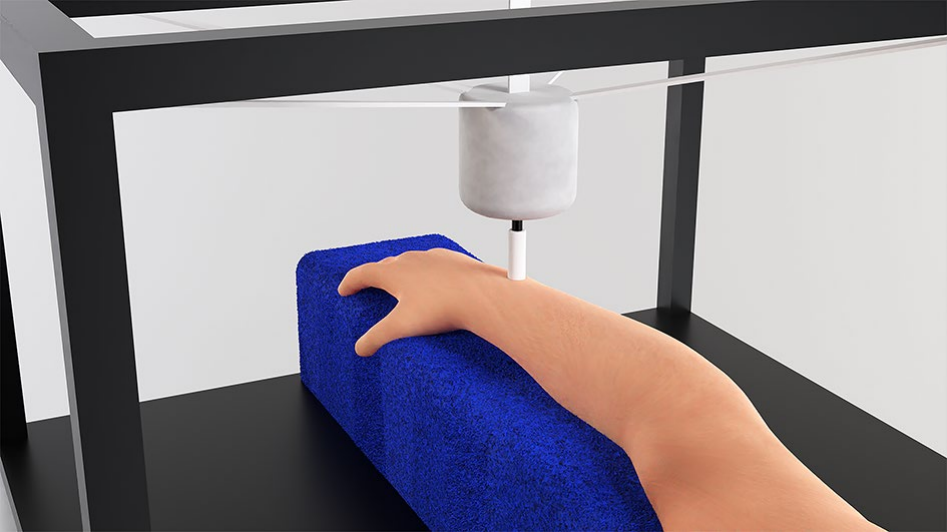
3D render of the EHS Research Group haptic stimulation rig, shown from the participant’s point of view, with the arm resting on a blue foam surface and the probe (attached to the shaker unit) contacting the centre of the dorsal wrist.

For the sentence identification task, the EHS Research Group haptic stimulation rig^10^ was used (shown in Fig. 7). This consisted of a Ling Dynamic Systems V101 shaker suspended from an aluminium strut frame by an adjustable elastic cradle. The shaker had a downward facing circular probe with a 10-mm diameter, which contacted the participant’s dorsal wrist. A foam block with a thickness of 95 mm was placed below the shaker probe for participants to rest their forearm on. The probe applied a downward force of 1N, which was calibrated using a B&K UA-0247 spring balance. The shaker was driven using a MOTU UltralLite-mk5 sound card, RME QuadMic II preamplifier, and HVLab Tactile Vibrometer power amplifier. The vibration output was measured using a B&K 4533-B-001 accelerometer and calibrated using a B&K type 4294 calibration exciter. All stimuli had a total harmonic distortion of less than 0.1%.

Masking noise in the experiment phase and speech audio in the familiarisation phase were played to the participant through Sennheiser HDA 300 headphones, driven by the MOTU UltralLite-mk5 sound card. The headphones were calibrated using a B&K G4 sound level meter with a B&K 4157 occluded ear coupler (Royston, Hertfordshire, UK). Sound level meter calibration checks were carried out using a B&K Type 4231 sound calibrator.

### Procedure

For each participant, the behavioural experiment was completed in a single session lasting approximately two hours. Participants first gave informed consent to take part in the study. They then completed a screening questionnaire, which ensured that they (1) had not had any injury or surgery on their hands or arms, (2) did not suffer from conditions that might affect their sense of touch, and (3) had not been exposed to intense or prolonged hand or arm vibration over the previous 24 h. Their self-reported hearing health was also recorded.

Following this, the participant’s skin temperature on the index fingertip of the dominant hand was measured. When the participant’s skin temperature was between 27 and 35 °C, their vibro-tactile detection thresholds were measured at the index fingertip following ISO 13091-1:2001^77^. During these measurements, participants applied a downward force of 2N, which was monitored by the participant and experimenter using the HVLab Vibro-tactile Perception Meter display. Participants were required to have touch perception thresholds in the normal range (< 0.4 m/s^−2^ RMS at 31.5 Hz and < 0.7 m/s^−2^ RMS at 125 Hz), conforming to ISO 13091‑2:2021^78^. The fingertip was used for screening as normative data was not available for the wrist. If all screening stages were passed, the participant’s wrist dimensions were measured at the position they would usually wear a wristwatch, and they proceeded to the experiment phase.

In the experiment phase, participants sat in front of the EHS Research Group haptic stimulation rig^10^. They placed their arm on the foam surface with the shaker probe contacting the centre of the dorsal wrist, at the position where they would normally wear a wristwatch. During the experiment phase, participants performed a two-alternative focused-choice sentence identification task, in which they were asked to select which of two alternative sentences had been presented through tactile stimulation. This was done using a custom-built MATLAB (2022b) app with three buttons, one that allowed the user to play the stimulus for the current trial, and two that displayed the sentence text alternatives for the current trial. The app also displayed the instruction: “Select the text that matches the sentence played through vibration”. When the play button was pressed, both the buttons displaying the sentence text for the alternative response options turned green for the duration of the stimulus. The sentence text buttons were only selectable after the stimulus had been played at least once.

Before testing began, participants completed familiarisation to ensure they understood the task. In this stage, English male and female speech was used that was different from that used in testing. The male talker was from the ARU speech corpus (ID: 02)^79^ and the female was from the University of Salford speech corpus^80^. They each spoke four different sentences from the Harvard sentences set^81^. Sentences were only played without background noise and with no noise reduction applied. The participant was permitted to feel the sentence through tactile stimulation or to hear the sentence through the headphones (without tactile signal processing). There was no limit placed on the number of times these sentences could be repeated and the participant was encouraged to ask the experimenter questions if they were unsure of the task. Once the participants selected one of the sentences, all buttons became inactive for 500 ms. During this period, green text reading “Correct” was displayed if the response was correct, or red text reading “Incorrect” was displayed if the response was incorrect. Once the experimenter confirmed that the participant understood the task, they continued to the testing stage.

During testing, the participant performed the same task as in familiarisation, except that they could not opt to hear the audio stimulus, and the headphones played masking noise. Participants were also limited to a maximum of four repeats of the tactile stimulus per trial, after which the play button became inactive, and they were forced to select one of the two sentence text alternatives. During testing, participants were presented with each of the sentence pairs shown in Table 2. The testing regime was designed to determine whether relevant acoustic contrasts in running speech could be accurately perceived via tactile stimulation, but to avoid floor effects due to the poor open-set intelligibility for tactile-only stimuli (without introducing extensive training or a larger number of sentence choices with high short-term memory load). All sentences were tested in all four experimental conditions once (in quiet and in background noise, both with and without the quasi-causal DPRNN). This meant that there was a total of 480 trials for each participant during the testing stage. The trial order was randomised for each participant, with a rule that the same sentence could not appear in consecutive trials.

The experimental protocol was approved by the University of Southampton Faculty of Engineering and Physical Sciences Ethics Committee (ERGO ID: 68477). All research was performed in accordance with the relevant guidelines and regulations.

### Statistics

#### Objective assessment

In the objective assessment, the effectiveness of the quasi-causal DPRNN was compared to a causal and non-causal DPRNN, as well as to the traditional log-MMSE method. In the first part of the assessment, after the envelopes were extracted using the tactile vocoder, the SI-SDR for speech in noise was calculated relative to the time-aligned envelopes for the clean speech signal (without noise reduction). The median score across the eight envelopes was taken for each of the 120 male and female sentences from the EHS Research Group Sentence Corpus. Analysis consisted of a four-way RM-ANOVA (see “Results”) followed by sixteen planned *t*-test, with Bonferroni-Holm multiple comparisons correction^82^ applied. These compared (1) the SI-SDRs for each noise reduction method to a baseline condition with no noise reduction applied, (2) the improvement in the SI-SDR from baseline for the male and female talkers with the quasi-causal DPRNN, and (3) the improvement in the SI-SDR with the quasi-causal DRPNN (for both talkers together) and the improvement for the causal DPRRN, the non-causal DPRNN, and the log-MMSE method. Data were determined to be normally distributed based on visual inspection as well as Kolmogorov–Smirnov and Shapiro–Wilk tests.

In the second part of the assessment, SI-SDR and eSTOI scores were computed for each audio sample (without tactile vocoding). For this assessment, the unprocessed clean speech audio was used as the reference.

#### Behavioural assessment

The percentage of correctly identified sentences was calculated for the male and female talkers in each condition. Primary analysis consisted of a three-way RM-ANOVA (see “Results”) and six two-tailed *t*-tests, with a Bonferroni-Holm multiple comparisons correction applied. The *t*-tests compared: (1) identification in quiet with and without noise reduction; (2) identification in noise with and without noise reduction; (3) identification in quiet without noise reduction and identification in noise with noise reduction; (4) identification in quiet and in noise, both without noise reduction; (5) the difference in identification in quiet with and without noise reduction for the male and female talkers; and (6) the difference in identification in noise with and without noise reduction for the male and female talkers. Data were determined to be normally distributed based on visual inspection and Kolmogorov–Smirnov and Shapiro–Wilk tests.

Following the primary analysis, exploratory secondary analysis was performed. These analyses were not corrected for multiple comparisons, as no effects were anticipated, following results from previous studies (e.g.,^10^).

## Data Availability

The datasets generated and analysed during the current study are available in the University of Southampton’s Research Data Management Repository at: 10.5258/SOTON/D3018.
